# Association Between Early Pregnancy Body Mass Index and Gestational Weight Gain in Relation to Neonatal Birth Weight

**DOI:** 10.7759/cureus.27089

**Published:** 2022-07-21

**Authors:** Ipsita Mohapatra, Nikku Harshini, Subha R Samantaray, Gitismita Naik

**Affiliations:** 1 Obstetrics and Gynecology, All India Institute of Medical Sciences, Kalyani, IND; 2 Obstetrics and Gynecology, Prathima Institute of Medical Sciences, Karimnagar, IND; 3 Community Medicine, All India Institute of Medical Sciences, Kalyani, IND

**Keywords:** body mass index, early pregnancy bmi, gestational weight gain, large for gestational age, small for gestational age (sga), iom recommendation, neonatal birth weight

## Abstract

Introduction: Maternal early pregnancy body mass index (BMI) and gestational weight gain (GWG) strongly correlate with pregnancy outcomes. Gestational hypertension and diabetes have been associated with overweight and obesity in pregnancy. A low pre-pregnancy BMI has been associated with low birth weight and preterm birth.

Method: This observational study was carried out from November 2018 to July 2020 in a tertiary care hospital in South India with a sample size of 100. Pregnant women with uncomplicated singleton pregnancies booked for regular antenatal care by 10 weeks of gestation were included in the study. During the participants' antenatal check-ups, detailed history and examinations were made. The weight of the participants was recorded at every antenatal check-up. Information about the gestational age at delivery and the birth weight of the neonates were collected following delivery.

Results: The mean age of women was 25.83 + 2.74 years. Of women who delivered low birth weight neonates (<2.5 kg), 86% had GWG below the Institute of Medicine (IOM) recommendation. A total of 57% of women with normal early pregnancy BMI and 67% of obese women had GWG within the IOM recommendation. Early pregnancy BMI had a positive correlation with neonatal birth weight (r (98) = 0.779, p = 0.001). Of the underweight pregnant women, 72% gave birth to small for gestational age (SGA) babies, and 97% percent of normal early pregnancy BMI women delivered neonates with normal weight for gestational age. A total of 33% of overweight and 50% of obese women had large for gestational age (LGA) babies.

Conclusion: Results from this study suggest that maternal early pregnancy BMI is more strongly associated with neonatal birth weight than GWG. Therefore, early pregnancy BMI may be an important focus for counseling during pregnancy.

## Introduction

Maternal early pregnancy body mass index (BMI) and gestational weight gain (GWG) have a meaningful impact on pregnancy outcomes. The birth weight of the neonate is the reflection of the various conditions during pregnancy and has an impact on the quality of life, the growth and development of the child, as well as childhood morbidity and mortality [1]. During the past few years, a secular trend toward increased birth weight and macrosomia related to greater maternal weight has been observed [2]. Early pregnancy overweight and obesity have been associated with adverse maternal outcomes [3]. Macrosomia increases the risk of cesarean delivery, shoulder dystocia, and subsequent childhood obesity [1,3].

Adequate intake of macronutrients and micronutrients during pregnancy promotes these processes, while undernutrition and overnutrition can be associated with adverse pregnancy outcomes [4-9]. Data from human epidemiologic studies suggest that maternal undernutrition impairs placental formation, leading to a reduction in placental size, alterations in histomorphology, and reduction of blood flow, which can diminish nutrient delivery to the fetus [10]. A low early pregnancy BMI has been associated with low birth weight (LBW) and preterm birth [3]. In addition to fetal growth restriction, maternal undernutrition has been associated with LBW and preterm delivery, plus the short- and long-term complications associated with these outcomes, and with maternal complications, such as life-threatening hemorrhage [11-14].

Thus, the incidence of pregnancy complications is higher at the upper and lower extremes of weight gain. Excessive GWG may also increase the risk of childhood obesity and maternal weight retention long after delivery. Excessive GWG in the first pregnancy is predictive of excessive weight gain in subsequent pregnancies [15]. Adding to this problem, women are more likely to gain rather than lose weight between pregnancies, and weight gain from one pregnancy to the next increases the risks of gestational diabetes mellitus, pregnancy-induced hypertensive diseases, cesarean delivery, preterm birth, large for gestational age (LGA), stillbirth, and cleft palate [16].

In 2009, a revised edition of the Institute of Medicine (IOM) guidelines for GWG was developed, advising the optimal GWG for women in different categories of BMI according to the WHO classification [11]. Limited information is available on the application of these IOM guidelines in the Indian population. In addition, there is a need to validate the IOM GWG guidelines in different populations. In India, most women come for their antenatal checkups in the late first trimester, and hardly any women come for a pre-conceptional check-up. So, even if pre-pregnancy BMI measurement has been advised by IOM, it is difficult to obtain these data in the rural population of India. Rather it is feasible to collect the early pregnancy BMI of the women attending the antenatal clinic.

From this perspective, the objective of this study was to investigate the association between early pregnancy maternal BMI and neonatal birth weight. In addition, it would also be possible to evaluate the extent to which women that are below, within, and above IOM GWG guidelines deliver normal birth weight infants, as previously done among other populations.

## Materials and methods

Study design, setting, and duration

This observational study was carried out from November 2018 to July 2020 in a tertiary care hospital in South India.

Aims and objectives

This study aimed to analyze the influence of early pregnancy BMI on neonatal birth weight and to study the association between GWG in pregnancy and neonatal birth weight.

Sample size and sampling strategy

A minimum sample size of 96 is considered using the following formula: n = Z_(1-α/2)_^2^p(1-p)/d^2^. Where Z_(1-α/2)_ = 1.96 at 95% confidence interval, p = expected prevalence of study condition in given population assumed to be 50% (as there was no previous relevant literature), and absolute precision (d) is taken as 10%, with the effect size of one. Considering the attrition rate of 10%, the sample size was adjusted to 107. The study was approved by the institutional ethics committee and review board. A convenient sampling method was used for the enrolment of participants after taking informed written consent.

Inclusion and exclusion criteria

Pregnant women with uncomplicated singleton pregnancies booked for regular antenatal care by 10 weeks of gestation were included in the study. Antenatal cases with ages less than 18 and more than 45 years, hyperemesis gravidarum, hypertension, endocrine disorders, multiple gestations, preterm delivery (before 37 completed weeks), or any medical or systemic illness complicating pregnancy were excluded.

Methodology

Data of the enrolled participants were collected in the pre-formed data collection sheet. The demographic data of the participants and their BMI were recorded during the booking visit (by 10 weeks) in the first trimester. Weight gain throughout pregnancy and weight in the first trimester (recorded at 11-12 weeks), second trimester (measured at 27-28 weeks), and third trimester (recorded at 36-37 weeks) were entered into the data collection sheet. Information about the gestational age at delivery and the birth weight of the neonate were collected from the case sheet following delivery. During the data collection period, seven pregnant women developed complications (four women developed pre-eclampsia, two women developed gestational diabetes mellitus, and one woman was lost to follow-up), and were excluded from the study. So, finally, 100 women were included in the analysis.

Operational definitions

The operational definitions used in the study are as follows: underweight = BMI less than 18.5 kg/m^2^; normal = BMI between 18.5 and 24.9 kg/m^2^; overweight = BMI between 25.0 and 29.9 kg/m^2^; obese = BMI more than 30.0 kg/m^2^; small for gestational age (SGA) = babies having birth weight below 2.5 kg; LGA = babies having birth weight above 3.5 kg; average for gestational age (AGA) = babies having a birth weight between 2.5 and 3.5 kg; GWG as per IOM guidelines = recommended weight gain is 12.5-18 kg for underweight, 11.5-16 kg for normal weight, 7-11.5 kg for overweight, and 5-9 kg for obese women.

Statistical analysis

All the data were collected and tabulated in a Microsoft Excel sheet (Microsoft Corporation, Redmond, WA) and data were analyzed with IBM SPSS Statistics version 22 (IBM Corp., Armonk, NY). Categorical data were represented as frequencies and percentages whereas continuous data were expressed as mean and standard deviation. The chi-square test or Fisher's exact test was used to compare the categorical data. Pearson correlation was used to assess the relationship between continuous variables. Univariate linear regression analysis was performed to determine the associations between birth weight and other covariables (i.e. maternal height, weight, early trimester BMI, and GWG). For all analyses, a p-value of less than 0.05 was considered statistically significant.

## Results

The relation between socioeconomic status and GWG is depicted in Table 1. The majority of cases (51%) belonged to the lower middle class and the upper middle class (27%). In a comparison of GWG within each class, 56.5% (13 out of 23) of women achieved weight gain below the IOM recommendations, and 46.6% (14 out of 30) of women achieving weight gain above the IOM recommendations were from the lower middle class. However, in the upper middle class, most of the women (48.14%) were above the IOM recommendations and no difference was observed in the lower class. Overall, the difference was not statistically significant (p > 0.05).

**Table 1:** Relation between socioeconomic status and gestational weight gain * p > 0.05. GWG (IOM): gestational weight gain as per Institute of Medicine recommendation.

Socioeconomic status*	GWG (IOM)			Total
	Above (n = 30)	Within (n = 47)	Below (n = 23)	
Upper middle	9	13	5	27
Lower middle	14	24	13	51
Lower	7	10	5	22

Table 2 depicts the descriptive statistics of the group. Among the pregnant women in the study, 46% were in the age group of 20-25 years, 49% were in the age group of 26-30 years, and 5% were older than 30 years. The mean age of women was 25.83 + 2.74 years, which ranged from 20 to 34 years, with 95% within 21-30 years. Mean weight gain was 1.46 ± 0.56 kg in the first trimester, 5.17 ± 1.11 kg in the second trimester, and 5.02 ± 1.19 kg in the third trimester. The mean total weight gain was 11.7 ± 2.3 kg. The mean birth weight was 2.3 ± 0.5 kg.

**Table 2:** Age and anthropometric characteristics

	Minimum	Maximum	Mean	SD
Age (years)	20	34	25.83	2.74
Height (meters)	1.56	1.68	1.61	0.03
Weight (kg)	40	94	63.23	12.28
BMI (1st trimester)	15.62	36.71	24.14	4.71
1st-trimester weight	41.0	95.0	64.69	12.29
1st-trimester weight gain	0	3.0	1.46	0.56
2nd-trimester weight	45.0	98.0	69.87	12.27
2nd-trimester weight gain	3.0	9.0	5.17	1.11
3rd-trimester weight	48.0	100.0	74.89	12.14
3rd-trimester weight gain	2.0	8.0	5.02	1.19
Total weight gain	6.0	17.0	11.7	2.3
Birth weight	2.0	4.1	2.3	0.5

In Table 3, the relationship between early pregnancy BMI and GWG as per the IOM recommendations has been made. The majority (57%) of women with normal BMI and obese (67%) women had GWG within the IOM recommendation. Whereas the majority of underweight (61%) and overweight (67%) women had GWG below and above the recommendations, respectively. The difference in distribution was found to be statistically significant (p < 0.05).

**Table 3:** Relation between early pregnancy BMI and gestational weight gain as per the IOM recommendations * p < 0.001. IOM: Institute of Medicine.

BMI group	Gestational weight gain (IOM)			Total*
	Above	Within	Below	
Underweight	0 (0%)	7 (39%)	11 (61%)	18
Normal	4 (11%)	21 (57%)	12 (32%)	37
Overweight	22 (67%)	11 (33%)	0 (0%)	33
Obese	4 (33%)	8 (67%)	0 (0%)	12
Total	30 (30%)	47 (47%)	23 (23%)	100

Table 4 shows the relation between neonatal birth weight and GWG as per the IOM recommendations. About 86% of women who delivered low birth weight neonates (<2.5 kg) belonged to GWG below the IOM recommendation. The majority (76.5%) of LGA neonates belonged to women with GWG above the IOM recommendation. This difference in distribution was found to be statistically significant (p < 0.05).

**Table 4:** Relation between gestational weight gain and neonatal birth weight as per the IOM recommendation Within the neonatal birth weight group, percentages are expressed in closed parenthesis. * Birth weight in kilogram; ** p < 0.001. IOM: Institute of Medicine; LGA: large for gestational age; SGA: small for gestational age; AGA: average for gestational age.

Gestational weight gain (IOM)	Neonatal birth weight*			Total**
	LGA (n = 17)	AGA (n = 69)	SGA (n = 14)	
Above	13 (76.5%)	17 (25%)	0 (0%)	30
Within	4 (23.5%)	41 (59%)	2 (14%)	47
Below	0 (0%)	11 (16%)	12 (86%)	23

Table 5 shows the relation between the different BMI groups and the neonatal outcome. Of underweight women, 72% gave birth to SGA babies. Of women with normal BMI, 97% delivered neonates with normal weight for gestational age. A total of 33% of overweight and 50% of obese women delivered LGA babies. This difference in distribution was found to be statistically significant (p < 0.05).

**Table 5:** Relation between early pregnancy BMI and birth weight Within the BMI group, percentages are expressed in closed parenthesis. * p = 0.001. LGA: large for gestational age; SGA: small for gestational age; AGA: average for gestational age.

BMI group	Neonatal birth weight			Total*
	LGA (n = 17)	AGA (n = 69)	SGA (n = 14)	
Underweight	0 (0%)	5 (28%)	13 (72%)	18
Normal	0 (0%)	36 (97%)	1 (3%)	37
Overweight	11 (33%)	22 (67%)	0 0%)	33
Obese	6 (50%)	6 (50%)	0 (0%)	12

The relation between early pregnancy BMI, GWG as per the IOM recommendations, and the neonatal outcome has been depicted in Table 6. All the cases in the LGA group had a BMI of either obese or overweight. The majority (13 out of 17) of women in the LGA group belonged to GWG above the IOM recommendation. None of the women in the LGA group belonged to GWG below the IOM recommendation. All the women in the SGA group had either normal or underweight BMI. The majority (12 out of 14) of women in the SGA group belonged to GWG below the IOM recommendation; in addition, none of them belonged to the GWG above the IOM recommendation.

**Table 6:** Relation between early pregnancy BMI, gestational weight gain, and birth weight * Statistically significant. GWG (IOM): gestational weight as per Institute of Medicine recommendation; LGA: large for gestational age; SGA: small for gestational age; AGA: average for gestational age.

Birth weight	Early pregnancy BMI	GWG (IOM)			Total	P-value
		Above (n = 30)	Within (n = 47)	Below (n = 23)		
LGA	Obese	4	2	0	6	0.482
	Overweight	9	2	0	11	
	Total	13	4	0	17	
AGA	Normal	4	21	11	36	0.001*
	Obese	0	6	0	6	
	Overweight	13	9	0	22	
	Underweight	0	5	0	5	
	Total	17	41	11	69	
SGA	Normal	0	0	1	1	0.672
	Underweight	0	2	11	13	
	Total	0	2	12	14	

In Table 7, a correlation has been made between the first trimester BMI, GWG of the antenatal women, and birth weight. A negative correlation was observed between BMI and GWG (r (98) = -0.159, p = 0.115). A positive correlation was observed between GWG and birth weight (r (98) = 0.163, p = 0.105). A strong positive correlation was noticed between BMI and birth weight (r (98) = 0.779, p = 0.001).

**Table 7:** Correlation between early pregnancy BMI, gestational weight gain, and birth weight ** Significant (p* *< 0.05).

Variable	Pearson correlation (r)	P-value
BMI with gestational weight gain	-0.159	0.115
Gestational weight gain with birth weight	0.163	0.105
BMI with birth weight	0.779	0.001**

To further explore the associations between birth weight and maternal anthropometric characteristics, a univariate linear regression analysis was performed. Table 8 shows that birth weight variance is predicted by maternal weight. The variances attributed to maternal BMI seem to be a function of maternal weight measured in the early first trimester itself. Correlation coefficients also show a high correlation with maternal weight, maternal weight per trimester, and maternal BMI.

**Table 8:** Association between birth weight and maternal anthropometric characteristics

Dependent	Independent	R	R^2^	Significance
Birth weight	Maternal BMI	0.779	0.607	0.000
	Maternal height (m)	0.128	0.016	0.205
	Maternal weight (kg)	0.754	0.568	0.000
	Maternal total weight gain during pregnancy	0.163	0.027	0.105
	Maternal 1st-trimester weight	0.758	0.575	0.000
	Maternal 2nd-trimester weight	0.606	0.602	0.000
	Maternal 3rd-trimester weight	0.793	0.630	0.000

## Discussion

In the present study, 100 pregnant women with uncomplicated singleton pregnancies booked at a tertiary care hospital by 10 weeks of pregnancy for antenatal care and delivering at term were studied during the period from August 2018 to July 2020.

In the study, the majority of women (51%) belonged to the lower middle class. Most of the women gaining weight outside the 2009 IOM recommendations for GWG were lower middle class but this difference was not statistically significant. Socioeconomic status may play a role in weight gain in pregnancy probably in terms of nutrition, knowledge about optimal care during pregnancy, and better adherence to nutritional supplements. However, a systematic review of 16 studies including 680,613 pregnant women concluded the relationship between socioeconomic status and GWG was inconsistent [17].

In our study, the prevalence of underweight, normal, overweight, and obese women was 18%, 37%, 33%, and 12%, respectively. Overall, the majority of cases in our study (47%) have attained GWG within the IOM recommendations, and 30% and 23% achieved GWG above and below the recommendation, respectively. On the contrary, a systematic review of 13 studies by Arora et al. reported that the majority of pregnant Indian women achieved less GWG than the recommendations whereas a mixed trend was noticed among the other Asian pregnant women [18]. Another systematic review, meta-analysis, and meta-regression of observational studies included 1,309,136 pregnant women. Goldstein et al. also reported the GWG of 47% and 23% above and below the IOM recommendation, which is similar to our study [19]. In another multicenter retrospective cohort study involving 48,867 primiparous women from Mainland China, Li et al. reported that only 36.8% of the women had a weight gain that was within the recommended range; 25% and 38.2% had weight gains that were below and above the recommended range, respectively [20]. Globally, the incidence of overweight and obesity in pregnancy is increasing but lower and middle-income countries like India also have a problem of low pre- and early pregnancy BMI.

In the present study, for the majority of women with normal early pregnancy BMI as well as obesity, the GWG was within the IOM recommendation, whereas most underweight and overweight women had GWG above and below the recommendation, respectively. In contrast, in a retrospective, observational study of 2728 pregnant women in South India, Bhavadharini et al. [21] reported a higher prevalence of early pregnancy overweight and obesity (18.5% and 46.9%, respectively). They also observed that the majority of women of all BMI categories gained weight less than recommended, whereas 28.5% of obese women gained more weight. We observed a mild negative correlation between early pregnancy BMI and GWG (r (98) = -0.159, p = 0.115), which was statistically insignificant. In a retrospective cohort study of 474 pregnant women, Nowak et al. reported no specific association between pre-pregnancy BMI and inadequate GWG but a high BMI correlated with excessive GWG (overweight = OR: 3.0, 95% CI: 1.84-3.87; obese = OR: 2.45, 95% CI: 1.1-5.48) [22].

In comparison, about 86% of mothers who delivered low birth weight neonates had GWG below the IOM recommendations. Of mothers who delivered LGA neonates, 76% had GWG above the IOM recommendations. This difference in distribution was found to be statistically significant (p < 0.05). We also observed a mild positive correlation between GWG and neonatal birth weight (r (98) = 0.163, p = 0.105). These findings were consistent with a retrospective cohort study by Du et al. [23], involving 3773 Chinese women, who observed that GWG, as well as pre-pregnancy BMI, was positively correlated with neonatal birth weight (p < 0.05). Similar to our study, Goldstein et al. [19] concluded that GWG below the recommendations was associated with a higher risk of SGA (OR: 1.53, 95% CI: 1.44-1.64), whereas GWG above the recommendations was associated with a higher risk of LGA (OR: 1.85, 95% CI: 1.76-1.95).

Of underweight women, 72% gave birth to SGA babies. Of women with normal early pregnancy BMI, 97% delivered neonates with normal weight for gestational age. A total of 33% of overweight and 50% of obese women delivered LGA babies. This difference in distribution was found to be statistically significant (p < 0.05). We also observed a good positive correlation between early pregnancy BMI and birth weight (r (98) = 0.779, p = 0.001). These results were consistent with a study done by Li et al. [24]. The authors observed a low pre-gestational BMI appeared as a risk factor for SGA (OR: 1.91, 95% CI: 1.47-2.50), and pre-gestational overweight/obesity (OR: 1.85, 95% CI: 1.58-2.17) and excessive GWG (OR: 1.87, 95% CI: 1.67-2.11) were both positively associated with the risks of LGA. These results are consistent with a study done by Nowak et al. [22]. In their study, the authors observed no statistically significant association between maternal pre-pregnancy BMI and the prevalence of SGA. However, underweight women with inadequate GWG showed a higher risk of bearing SGA babies (OR: 5.2, 95% CI: 1.57-17.18), whereas high BMI correlated with excessive GWG (overweight = OR: 3.0, 95% CI: 1.84-3.87; obese = OR: 2.45, 95% CI: 1.1-5.48).

Limitations of the study

One major limitation of this study is the small sample size. Pre-pregnancy is the best time for counseling, but most antenatal cases report only in the late first trimester, which may be one of the limiting factors. Also, uniformity in estimating the BMI at a particular gestational age before 10 weeks could not be maintained. There is also a possibility of bias due Hawthorne effect, where patients became more alert because of their involvement in the study during their later gestational period.

## Conclusions

Results from this study suggest that maternal early pregnancy BMI is more strongly associated with neonatal birth weight than GWG. Therefore, early pregnancy BMI may be an important focus for counseling during pregnancy. Healthcare providers taking care of pregnant women should record the woman’s BMI at the initial antenatal visit and explain to her the advantages of the correct amount of weight gain, diet, and exercise, and, most importantly, the need to limit excessive weight gain to have the best outcome of pregnancy.
